# Herbivore-induced chemical and molecular responses of the kelps *Laminaria digitata* and *Lessonia spicata*

**DOI:** 10.1371/journal.pone.0173315

**Published:** 2017-03-02

**Authors:** Andrés Ritter, Léa Cabioch, Loraine Brillet-Guéguen, Erwan Corre, Audrey Cosse, Laurence Dartevelle, Harold Duruflé, Carina Fasshauer, Sophie Goulitquer, François Thomas, Juan A. Correa, Philippe Potin, Sylvain Faugeron, Catherine Leblanc

**Affiliations:** 1 Sorbonne Universités, UPMC University Paris 06, CNRS, UMR 8227, Integrative Biology of Marine Models, Station Biologique, Roscoff, France; 2 Centro de Conservación Marina and CeBiB, Facultad de Ciencias Biológicas, Pontificia Universidad Católica de Chile, Santiago, Chile; 3 Sorbonne Universités, UPMC University Paris 06, CNRS, FR2424, Analysis and Bioinformatics for Marine Science, Station Biologique, Roscoff, France; 4 Sorbonne Universités, UPMC University Paris 06, CNRS, UMI 3614, Evolutionary Biology and Ecology of Algae, Station Biologique, Roscoff, France; Heidelberg University, GERMANY

## Abstract

Kelps are founding species of temperate marine ecosystems, living in intertidal coastal areas where they are often challenged by generalist and specialist herbivores. As most sessile organisms, kelps develop defensive strategies to restrain grazing damage and preserve their own fitness during interactions with herbivores. To decipher some inducible defense and signaling mechanisms, we carried out metabolome and transcriptome analyses in two emblematic kelp species, *Lessonia spicata* from South Pacific coasts and *Laminaria digitata* from North Atlantic, when challenged with their main specialist herbivores. Mass spectrometry based metabolomics revealed large metabolic changes induced in these two brown algae following challenges with their own specialist herbivores. Targeted metabolic profiling of *L*. *spicata* further showed that free fatty acid (FFA) and amino acid (AA) metabolisms were particularly regulated under grazing. An early stress response was illustrated by the accumulation of Sulphur containing amino acids in the first twelve hours of herbivory pressure. At latter time periods (after 24 hours), we observed FFA liberation and eicosanoid oxylipins synthesis likely representing metabolites related to stress. Global transcriptomic analysis identified sets of candidate genes specifically induced by grazing in both kelps. qPCR analysis of the top candidate genes during a 48-hours time course validated the results. Most of these genes were particularly activated by herbivore challenge after 24 hours, suggesting that transcriptional reprogramming could be operated at this time period. We demonstrated the potential utility of these genes as molecular markers for herbivory by measuring their inductions in grazed individuals of field harvested *L*. *digitata* and *L*. *spicata*. By unravelling the regulation of some metabolites and genes following grazing pressure in two kelps representative of the two hemispheres, this work contributes to provide a set of herbivore-induced chemical and molecular responses in kelp species, showing similar inducible responses upon specialist herbivores in their respective ecosystems.

## Introduction

Brown algae are photosynthetic sessile macro-organisms of high ecological relevance in coastal ecosystems of temperate and polar regions. They belong to the Stramenopile lineage, which diverged early in the evolution from Opistokonts (including animals, fungi) and Plantae (land plants, green and red algae) and constitute one of the rare eukaryotic lineages to reach complex multicellularity [1]. Little is known about their biology, as they were mostly studied for their taxonomic and ecological diversity. The recent genome sequencing of the filamentous brown alga *Ectocarpus siliculosus* underlined particular molecular features in these organisms to cope with highly variable tidal environment such as an extended set of light-harvesting and pigment biosynthesis genes, and original metabolic processes such as halide metabolism [2]. Brown algae include mostly marine species ranging from small filamentous organisms to large kelps measuring several meters in size. Kelps (order of Laminariales) have primordial roles in rocky coastal ecosystems of temperate regions, from Northern and Southern hemispheres, as they form true forests of considerable biomass, hosting highly diversified ecosystems [3]. These marine organisms are also exploited for nutritional or industrial purposes and therefore constitute a significant economical resource. One particularly important question is what makes the ecological and evolutionary success of these giant marine primary producers. Indeed, kelps are continuously submitted to biotic attacks including viruses, bacteria, fungi, brown algal endophytes or grazing herbivores. Yet, they are the only macroalgal taxon that evolved large sizes (i.e. few m to tens of m), somehow escaping or overriding the effect of these attacks. The question is relevant also for the future of the rapidly growing field of kelp aquaculture. Indeed, as it is not possible to spread pesticides in the ocean (i.e. the currents would wash away the products before they actually have any effect in a marine culture), it is necessary to explore methods of protection against herbivores, pests and pathogens that rely on the activation of defense mechanisms by the seaweed itself.

Among biotic pressures, herbivore grazing has a major impact in marine benthic ecosystems [4]. To respond to these challenges, kelps are likely to develop adaptive strategies based on induced metabolic responses [5,6]. Most of the knowledge comes from studies on the North Atlantic kelp *Laminaria digitata*. This organism recognizes biotic threats by the perception of molecular cues such as oligoalginates, recognized as cell wall degradation fragments or lipopolysaccharides related to bacterial pathogens [7,8]. These molecules elicit a signaling cascade resulting in molecular defense responses [8,9]. Elicitor perception generates an early oxidative burst followed by the release of polyunsaturated free fatty acids that are subsequently oxidized into lipophilic signaling molecules grouped under the term oxylipins. In an evolutionary context, this innate immune response in kelps is reminiscent to animals and land plants mechanisms, where oxylipins play pivotal roles as stress-related signaling molecules. Animals predominantly synthesize eicosanoid (C20) derived oxylipins such as prostaglandins, thromboxanes, prostacyclins, leukotrienes or hydro(pero)xy-FA whereas land plants synthesize octadecanoid (C18) derived oxylipins including hydro(pero)xy-FA, divinyl ethers, volatile aldehydes or jasmonic acid and its derivatives (JAs) [10,11]. In contrast to plants, brown algae have conserved the capability of synthesizing eicosanoid fatty acids. As a consequence, these organisms synthesize a wide variety of oxylipins such as “animal-like” eicosanoid derived hydro(peroxy)des, epoxides and prostaglandins or “land plant-like” octadecanoid derived aldehydes and the JA precursors 13-HPOTrE or 12-OPDA [12–14]. The synthesis of oxylipins in brown algae is concomitant with the activation of defense responses at the transcriptional and metabolite level [6, 9, 10]. Moreover, previous results show that the kelp *L*. *digitata* can recognize the land plant oxylipin, MeJA that triggers defense reactions resulting in the establishment of resistance against invasion by its brown algal endophyte *Laminariocolax tomentosoides* [15]. Furthermore, recent analyses demonstrated the existence of elicitor-triggered systemic signals in *L*. *digitata* [16]. Localized oligoguluronate treatment in this kelp generates a systemic signal that induces a distant defense response against the blue limpet *Patella pellucida*. Interestingly, Chlorpromazine treatment (phospholipase inhibitor) could impair systemic signaling in *L*. *digitata*, suggesting the implication of FFA oxidation cascade in this process [16].

During interaction with herbivores, induced anti-grazing defenses have already been reported for most marine macroalgae, including Laminariales [17,18]. However, up to recently, these field or laboratory-controlled experiments were mainly based on herbivore feeding behavior, and few studies have yet explored the molecular and chemical bases of these inducible defense responses in brown algae, contrary to land plants. For kelps, although previous studies set up the basis for the investigation of kelp elicitor-induced defense responses, very few is known about metabolic regulations occurring during grazer attacks. In this context, we carried out metabolome and transcriptome analyses of the South Pacific kelp *Lessonia spicata* and the North Atlantic kelp *Laminaria digitata* challenged with their limpet specialist herbivores to infer common and specific responses in two kelp families (Lessoniaceae and Laminariaceae).

## Materials and methods

### Algal material

Juvenile sporophytes of *Laminaria digitata* (Hudson) J.V. Lamouroux (i.e. 5 cm long) and *Lessonia spicata* (Suhr) Santelices (i.e. 20–40 cm long) were collected from the surrounding shores near the Station Biologique of Roscoff (SBR)–France, at the Bloscon site (48°43’31”N; 3°58’8”O), and at the Estación Costera de Investigaciones Marinas (ECIM), Las Cruces—Chile (33°30’09”S: 71°37’59”O), respectively, and maintained at 14°C in running or filtered seawater (FSW) for 1–3 days before use. Their associated specialized herbivores, *Patella pellucida* Linnaeus in SBR and *Scurria scurra* in ECIM were collected at the same time under kelp beds and kept separately under running seawater until the experiment.

### Ethics statement

Relevant permissions were obtained for observational and material sampling from the French governmental authorities at Department of Maritime Affairs of Quimper (DDTM29) and sampling permits in Chile were obtained from the Director of the ECIM Las Cruces, who is responsible for the conservation of the Marine Coastal Protected Area of Las Cruces.

### Controlled grazing experiments

Grazing experiments consisted in co-incubating kelp individuals with their respective grazers: 3 individuals of *P*. *pellucida* for each *L*. *digitata* in 50 mL FSW filled Petri dish and five individuals of *S*. *scurra* for each *L*. *spicata* in 10 L of running seawater aquarium at 17°C. Grazing pressure lasted from 6, 12, 24 and 48 hours (3–4 replicates per incubation time, per species). Three to four replicates of sporophytes maintained in absence of limpets in similar experimental conditions were considered as controls for each incubation time. Kelp tissues were then frozen in liquid nitrogen and stored at -80°C.

### Metabolic profiling analyses

Metabolic extracts were obtained from 100 mg of frozen algal powder of *L*. *digitata* (n = 4 except for grazed-48h n = 2) or *L*. *spicata* (n = 3 except for control-48h n = 2) samples collected after 6h, 12h, 24h and 48h, with 1 mL MeOH:H_2_O (8:2), as already described [19]. For untargeted metabolomic analyses, 1.25 μg of 12-OH-lauric acid was added in each sample, as internal standard. After extraction, aliquots of 50 μL were separated by ultrahigh-pressure liquid chromatography (UPLC) and analyzed by mass spectrometry (MS) on a Thermo Scientific LTQ-Orbitrap Discovery^™^ mass spectrometer (Thermo Scientific) equipped with an Electro Spray Ionization (ESI) source running on the negative mode, as described [19]. Samples were separated using an Acclaim RSLC 120 C18 1.9 μm (2.1 x 100 mm) column 545 (Dionex; Thermo Fisher Scientific, Courtaboeuf, France) maintained at 20°C using 5 μL injection volume and a flow-rate of 250 μL.min^-1^ and the mobile phase (0.2% acetic acid in water–acetonitrile) was programmed from 95: 5 to 5: 95 acetonitrile–water (v: v).

Mass spectra data were processed by XCMS using the online version of Galaxy-Workflow4metabolomics [20], after conversion of raw spectra to mzXML format. Data processing was performed using centWave method for the peak picking, with a maximum deviation of 4 ppm. The signal/noise threshold was fixed at 10, the prefilter at 3,100 and the noise filter at 5000. For the first group step, density method was used, with the band width set at 30 and the minimum fraction of samples necessary at 0.7. For correction of retention time, the obiwarp method was used and a step size of 0.1 m/z. The second group step was performed using density method and a band width of 10. Fillpeaks step was used with the chrom filling method. Finally, annotation by CAMERA was set using a max ion charge of 2, a general ppm error of 5 and a precision of 4 decimals of m/z values. Areas for all peaks were normalized using that of the internal standard in the same sample, and normalized areas were used as ion relative abundances. The multivariate data analysis of relative ions abundances using Partial Least Squares—Discriminant Analysis (PLS-DA) was performed to test for differences in metabolite profiles among grazed and control samples. Further statistical multiple testing analyses were performed to identify the different ions, based on Student T-test and FDR-adjusted p-values (*P*). The analysis was carried out on Pareto scaling and log10-transformed data using the software SIMCA (13.0, Umetrics, Umeå, Sweden).

For amino acid, free fatty acid and oxylipin targeted profiling in *L*. *spicata* samples (n = 3 except for control-48h n = 2), the same extraction and quantification process was applied as for the untargeted analysis (see above), adding 1.25 μg of 12-OH-lauric acid and 10 μg of atropine in each sample, as internal standards for negative or positive ionization mode analyses, respectively. Free fatty acid and oxylipin profiles were obtained through negative mode LCMS analyses, and amino acid measurements in positive mode. The Hierarchical Clustering analysis of relative abundances changes was carried out using Pearson Correlation values in Multi Experiment Viewer 4.9 [19]. Statistical Kruskal-Wallis tests were then performed for each metabolite with grazing treatment and time as factors, using the free software R version 3.2.5.

### cDNA library sequencing and data analysis

Total RNA was extracted from 100 mg of frozen algal materials according to Apt et al. [21], treated with RNAse-free DNAse I (Stratagene, La Jolla, CA, USA) to eliminate genomic DNA contamination, and quantified using a Nanodrop ND 1000 spectrophotometer (Labtech International Ltd, Lewes, UK). For each species, total RNA was extracted from three independent biological replicates of control or grazed algal samples, collected after 6, 12, 24 and 48 hours for *L*. *digitata* (Ld) and after 6, 12 and 24 hours for *L*. *spicata* (Ls). For cDNA library sequencing, two pools of RNA, corresponding to Control or Grazed treatments for each species, were prepared by pooling the same amount of RNA samples extracted at each time point from independent biological triplicates, i.e. 12 RNA samples for Ld and 9 RNA samples from Ls. The four non-normalized shot-gun cDNA libraries, namely Ld-Control, Ld-Grazed, Ls-Control and Ls-Grazed, were constructed using the SMART cDNA library kit from Clontech (CA, USA), differentially- tagged with a specific adaptor and sequenced using 454 sequencing technology (Roche, Branford, CT, USA) at the Max Planck Institute of Berlin-Dahlem.

Raw sequencing data were deposited in the SRA database at NCBI (accessions SRR4149283 for Ld-Control, SRR4159455 for Ld-Grazed, SRR4240918 for Ls-Control, SRR4240919 for Ls-Grazed sequences). Raw reads were cleaned by removing adaptor sequences, empty reads and filtering reads with poor quality using SeqClean (https://sourceforge.net/projects/seqclean/files/). The cleaned 454-generated reads constituted the Expressed Sequence Tags (ESTs). For each species, all ESTs derived from Control and Grazed libraries were assembled together into non-redundant unigenes using Newbler v2.0.01.14 (http://www.my454.com), by including cDNA sequences of the two species available in public databases, to increase the quality of clustering. The unigenes sequences are available upon request. Each EST, belonging to Control or Grazed libraries, was associated to one unigene sequence (contigs or singletons). In order to compare the relative abundance of EST per unigene between the two libraries, we calculated their relative abundance with the following formula: Relative EST abundance of Unigene U in Library Lib = number of EST mapped to Unigene U in Library Lib * 1/(Total number of ESTs in Library Lib * Length of Unigene U (bp)).

Putative differentially-expressed unigenes were further identified according to their relative EST abundance into the two libraries, using IDEG6 web tool [22], based on concordant results from four different statistical tests, namely Pairwise Audic & Claverie, Pairwise Fisher Exact, Pairwise Chi-squared and Stekel & Falciani R tests [23]. The putative function of these differentially-expressed sequences was analyzed using Blastx and Blastn against the nr and nt databases (March 2016).

### Validation of differentially-expressed genes by qPCR analysis

To validate the putative molecular markers of grazing pressure, total RNA was extracted from 100 mg of frozen algal material of *L*. *digitata* or *L*. *spicata* sampled during laboratory-controlled grazing kinetics or in natural populations. For *L*. *digitata* grazing laboratory kinetics, the RNA samples (n = 3 for each time point) were those later pooled for EST analyses, whereas for *L*. *spicata*, a new experimental grazing experiment was conducted up to 48 hours, corresponding to a new set of RNA samples for both control and grazed algae (n = 3 for each time point). For *in situ* survey, grazed and un-grazed fronds from adult sporophytes of *L*. *digitata* and *L*. *spicata* (n = 7–8) were sampled at the same locations at Roscoff and Las Cruces (see above and S1 Fig). Field samples were immediately flash frozen in liquid nitrogen and kept at -80°C before RNA extraction. Gene expression was then analyzed by qPCR as described by Cosse et al. [9], using the primers sequences provided in S1 Table. Gene transcript level was normalized to the geometrical mean of two reference gene transcript levels in the same sample (tubulin and EF1a genes for *L*. *digitata*; EF1a and RPL36 genes for *L*. *spicata*). The Hierarchical Clustering analyses of gene expression changes was carried out on Pearson Correlation values in Multi Experiment Viewer 4.9 [19]. A two-way analysis of variance (ANOVA) with grazing treatment and time as categorical predictors was applied to qPCR expression data. All tests were performed using Statistica 7 (Statsoft, Tulsa, OK, USA).

## Results

### Specialist herbivores induced large metabolome alterations in both Chilean and European kelp species

**Fig 1:**
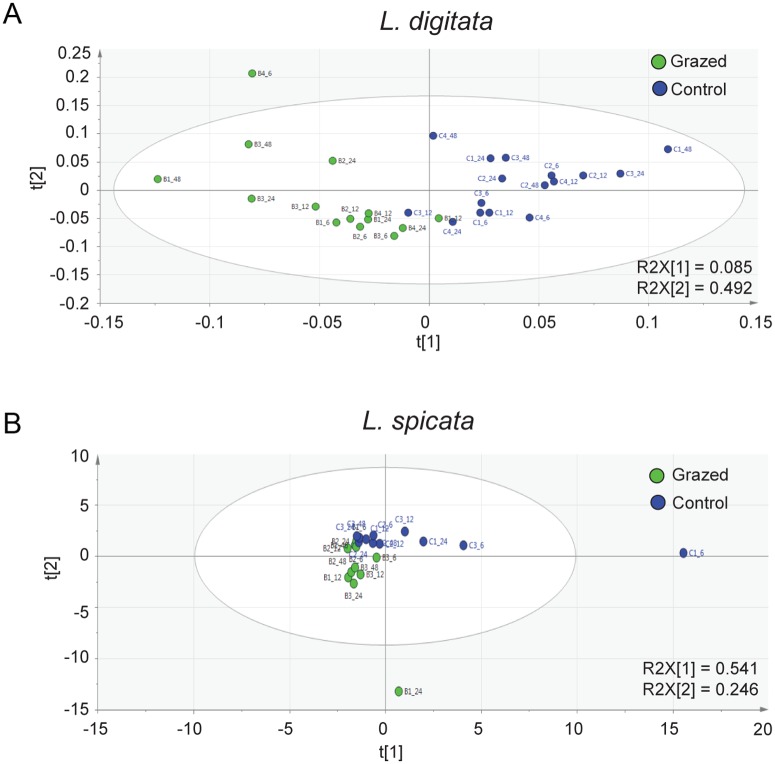
Specialist herbivores induced large metabolite variations in kelps. PLS-DA score plots of metabolite ions detected by LC-MS in *L*. *digitata* (**A**) and *L*. *spicata* (**B**) juveniles under controlled conditions (211 and 818 monoisotopic peaks or ions, respectively). Blue spots represent control algae and green spots represent grazed algae.

Metabolomic profiling of grazed algae by LC-MS in the negative mode provided 211 ion peaks in *L*. *digitata* and 818 in *L*. *spicata*, that were considered for multivariate analysis (S2 and S3 Tables). Partial least squares discriminant analysis (PLS-DA) of these datasets for *L*. *digitata* explained ~50% of the observed variation. In particular, the axis t[1] discriminated two groups of grazed and control individuals along t[2] axis (Fig 1A). For *L*. *spicata* datasets, PLS-DA analysis explained 80% of the observed variation. Grazed and controlled individuals were distinctively grouped and separated by t[2] axis that explained 54% of the observed variation (Fig 1B). Although multivariate metabolome analysis succeeded to discriminate between control and grazed kelps, PLS-DA analyses failed to distinguish a general pattern according to time in both species. This result suggests the establishment of a metabolic response from 6h of grazing exposure, which would persist over time of the experiment until 48h. Moreover only a small proportion of ions showed a significant change between the two treatments (T-test and FDR tests, *P*<0.01, 12 ions over 211 ions for *L*. *digitata* and 15 over 818 ions for *L*. *spicata*; see S4 and S5 Tables).

### Metabolic profiling of grazed *L*. *spicata* highlighted distinctive accumulation patterns of Amino Acids and Free Fatty Acids

**Fig 2:**
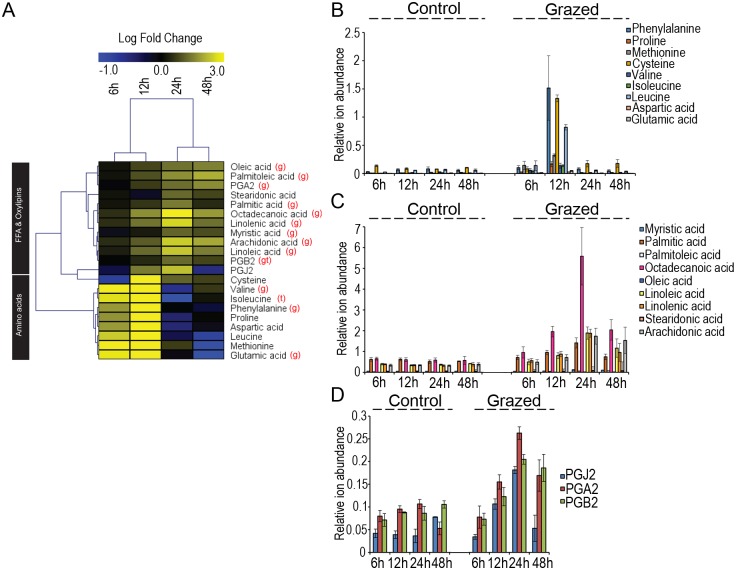
Metabolic profiling in grazed *L*. *spicata* highlighted distinctive accumulation dynamics of Amino Acids and Free Fatty Acids. (A) Heat map of the amino acids, free fatty acids and oxylipins detected in *L*. *spicata* under grazing pressure in laboratory-controlled conditions. Values represent the average log fold-change compared to control conditions (n = 3). Time points and compounds were clustered according to Pearson Correlation. The letters (g) and (t) indicate significant effects of grazing and time, respectively (*P*<0.05, Kruskal-Wallis test). (B-D) Time course of relative abundances of free amino acid (B), free fatty acid (C) and oxylipin contents (D) in control and grazed *L*. *spicata* (mean of three replicates ± SE).

We previously demonstrated the regulation of free fatty acid (FFA), oxylipin and amino acid (AA) metabolisms by biotic and abiotic stress in brown algae [7,12,19]. Accordingly, we profiled these metabolites to evaluate their possible functions in grazing defense. Unfortunately the resolution of *L*. *digitata* dataset was not sufficient to proceed to these targeted analyses all along the experimental time-course. We therefore carried out this analysis only in *L*. *spicata* which provided a highly resolved ion dataset. Indeed LC-MS analysis of extracts obtained from grazed and control individuals of *L*. *spicata* identified nine FFA, three oxylipins and nine AAs. Hierarchical Clustering Analysis of metabolite accumulation in grazed algae compared to controls made a clear distinction between early harvested samples at 6h and 12h opposed to late harvested samples at 24h and 48h (Fig 2A). Moreover, statistical analysis showed a significant induction effect of grazers (Kruskal-Wallis test; *P<0*.*05*) for 13 of the 21 profiled metabolites (Fig 2A; S6 Table).

Free AAs accumulated early upon grazing, with a 17-fold increase in total detected AA content after 12h of grazing compared to control. This was followed by a drastic drop in AA content, reaching control levels on longer grazer exposure from 24h onwards (Fig 2A and 2B). Specifically, 12h of grazing pressure increased concentration of phenylalanine (22-fold) and of methionine (34-fold) and its precursors, cysteine (16-fold) and aspartate (16-fold). The AA proline (14-fold) and its precursor glutamate (16-fold), but also valine (14-fold), leucine (15-fold) and isoleucine (9-fold) showed a similar pattern of induction with a peak after 12 hours of grazing exposure compared to controls (Fig 2B).

In contrast to AAs, FFA and FA-derivatives accumulated at latter time, and 10 out of the 12 profiles presented a significant induction by grazing exposure (Fig 2A; S6 Table). This difference in timing was clearly observed in the Hierarchical Clustering Analysis of metabolite accumulation (Fig 2A). Most of the detected FFA and FA-derivatives accumulated from 12 hours of grazing to peak at 24h and then dropped at 48h (Fig 2C and 2D). Remarkably, grazers induced a strong liberation of FFAs from both octadecanoid and eicosanoid pathways. Octadecanoic acid (C18:0) was the most highly accumulated FFA upon grazing exposure showing 10-fold increase at 24h (Fig 2A and 2C). Linoleic (C18:2), linolenic (C18:3) and arachidonic (C20:4) acids were the most highly accumulated desaturated FFAs and showed increases of 5–6 folds at 24h of grazing challenge compared to control conditions (Fig 2A and 2C). These FFAs are direct precursors of oxylipins. Accordingly, we detected significant accumulations of the cyclopentenone prostaglandins J2, A2 and B2 under grazing pressure. These oxylipins co-accumulated with their precursor arachidonic acid, increasing after 12h of grazing, reaching a peak at 24h and then decreasing down to control levels at 48h (Fig 2A and 2D).

### EST analysis and qPCR validation identified differentially expressed genes under grazing challenge in kelps

**Fig 3:**
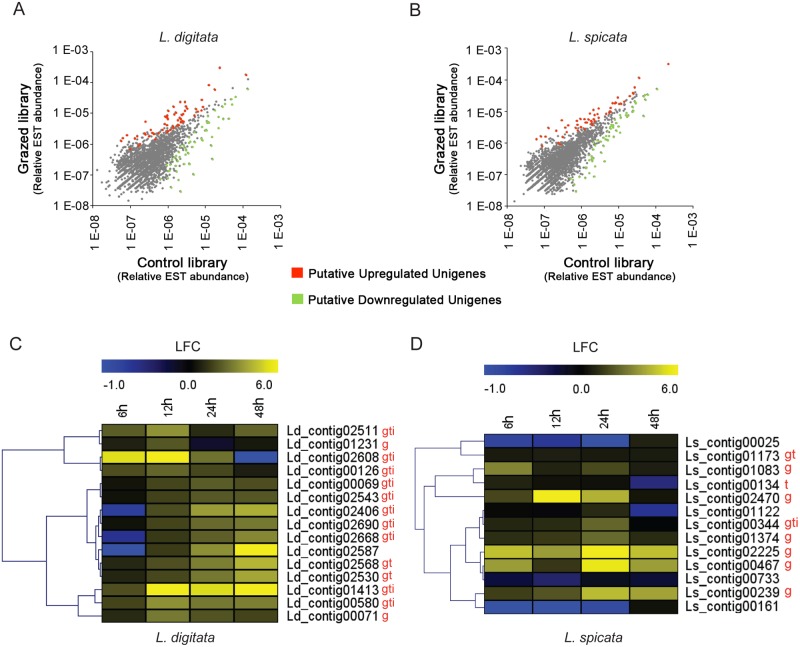
EST libraries combined to qPCR analyses of grazed *L*. *digitata* and *L*. *spicata* revealed differentially expressed transcripts. (**A,B**) Scatter-plot representations of relative EST abundance per unigene in Grazed and Control libraries of *L*. *digitata* and *L*. *spicata*. For calculation of Relative EST abundance, see Methods. Red and green dots respectively represent identified putative up- or down-regulated unigenes in grazed compared to control conditions. (**C,D**) Heatmaps showing the qPCR validation of grazing markers in laboratory controlled conditions. The expression pattern of selected unigenes are presented according to a hierarchical clustering analysis. Values are represented as log fold-change (LFC) of grazed vs control conditions (n = 3). The letters "g", "t" and "i" indicate significant effects of grazing, time and their interaction, respectively (*P*<0.05, 2-way ANOVA).

We next examined transcriptome variations of grazed *L*. *digitata* and *L*. *spicata* to identify particular genes or processes potentially related to herbivory in kelps. In this purpose, juveniles of *L*. *digitata* and *L*. *spicata* were challenged or not with their respective specialist grazers and collected after 6, 12, 24 and 48 hours for *L*. *digitata* and after 6, 12 and 24 hours for *L*. *spicata*. Triplicate RNA samples from each time point were pooled to generate four different cDNA libraries (one Control and one Grazed for each species) that were subsequently sequenced using 454 sequencing. A total of 444,474 reads were produced with an average length of 184 nucleotides (Table 1). After assembling together all ESTs (Expressed Sequence Tags) by species, 15,454 and 16,511 unigenes (singletons + contigs) were retrieved for *L*. *digitata* and *L*. *spicata*, respectively. Relative EST abundances per unigene in grazed and control libraries were compared using four different statistical tests to identify differentially-expressed candidate unigenes. Since libraries are composed of pooled RNAs without replication, the design has limited statistical power and therefore results must be carefully considered. Our analysis identified only a small fraction (0.8%) of unigenes (122 and 132 for *L*. *digitata* and for *L*. *spicata*, respectively) showing significant (*P*<0.01) differential expression between grazing and control treatments. (Fig 3A; S7 and S8 Tables). Namely, 64 unigenes showed significant up-regulation and 58 unigenes were down-regulated in grazed *L*. *digitata* (S7 Table). *L*. *spicata* analysis resulted in 64 up-regulated and 70 repressed unigenes upon grazing (S8 Table).

**Table 1:** General features of 454-generated ESTs libraries.

	*Laminaria digitata*	*Lessonia spicata*
# Total reads	201,664	242,810
# Total ESTs (after cleaning)	104,479	126,002
# ESTs in Control library	44,343	57,979
# ESTs in Grazed library	58,590	67,950
# cDNA seq. from NCBI	1,546	73
# Unigenes (after assembling)	15,454	16,511
Size range (nt)	50–2,817	50–1,335
Average length (nt)	187	181
# Contigs	2,676	2,716
Average length (nt)	358	361
# Singletons	12,778	13,795
Average length (nt)	151	146

Blastx and Blastn analysis of putative differentially expressed sequences against the nr database retrieved few homology with gene databases, underlying their probable specificity to brown algal sequences (S7 and S8 Tables). In many cases (i.e. 97/122 for *L*. *digitata* and 84/132 for *L*. *spicata*) Blast results retrieved non coding sequences, likely corresponding to 3’UTR sequences of the cDNA library. Indeed the brown algal genes have been shown to feature very long 3’UTR [2], leading to their over-prevalence in cDNA libraries.

To confirm the results obtained from *in silico* analyses of EST libraries, we selected 15 unigenes in *L*. *digitata* and 13 in *L*. *spicata* among the top putative up-regulated expressions under grazer pressure (S7 and S8 Tables). Expression patterns of these unigene candidates were followed in juvenile individuals of both species challenged with their specific limpet grazers *P*. *pellucida* and *S*. *scurra*. Triplicate independent samples of both species were harvested after 6, 12, 24 and 48 hours of grazing challenge for RNA extraction. The qPCR expression analysis in *L*. *digitata* showed significant induction effect (2-way ANOVA; *P<0*.*05*, n = 3) of grazer challenge in 14 out of the 15 tested unigenes (Fig 3B; S9 Table). The 2-way ANOVA test also found a significant effect (*P<0*.*05)* of time for 12 tested unigenes. Furthermore 10 tested unigenes presented significant interactions between time and grazing factors (*P<0*.*05)*, meaning that their up-regulation was dependent on a particular time (S9 Table).The relation between grazing and time was represented by a hierarchical clustering analysis of unigene fold-change expression of grazed compared to control algae in Fig 3B. This analysis identified a large group of 10 unigenes showing an expression pattern of induction at 24 to 48 hours of grazing pressure. The remaining 5 unigenes showed rather rapid induction, or significant downregulation.

We used the same validation approach in *L*. *spicata* by testing 13 unigenes that showed strong induction by grazer action in the EST library. This analysis identified 8 out of 13 unigenes as significantly different (2-way ANOVA; *P<0*.*05*, n = 3) between control and grazed groups (Fig 3C; S10 Table). In less cases than for *L*. *digitata*, 2-way ANOVA test also found some significant effects (*P<0*.*05)* of time and its interaction with grazing treatment, suggesting that the induction of one gene is dependent on a particular timing. Hierarchical clustering analysis grouped together 6 out of the 13 genes being significantly affected by grazing, time and the interaction between these factors (namely Ls_contig02470, Ls_contig00344, Ls_contig01374, Ls_contig02225, Ls_contig00467, Ls_contig00239, Fig 3C). These genes showed expression patterns increasing from 6h to peak at 24h of grazing pressure following a drop of induction at 48h (Fig 3C).

### Field validation of grazing responsive-marker genes

**Fig 4:**
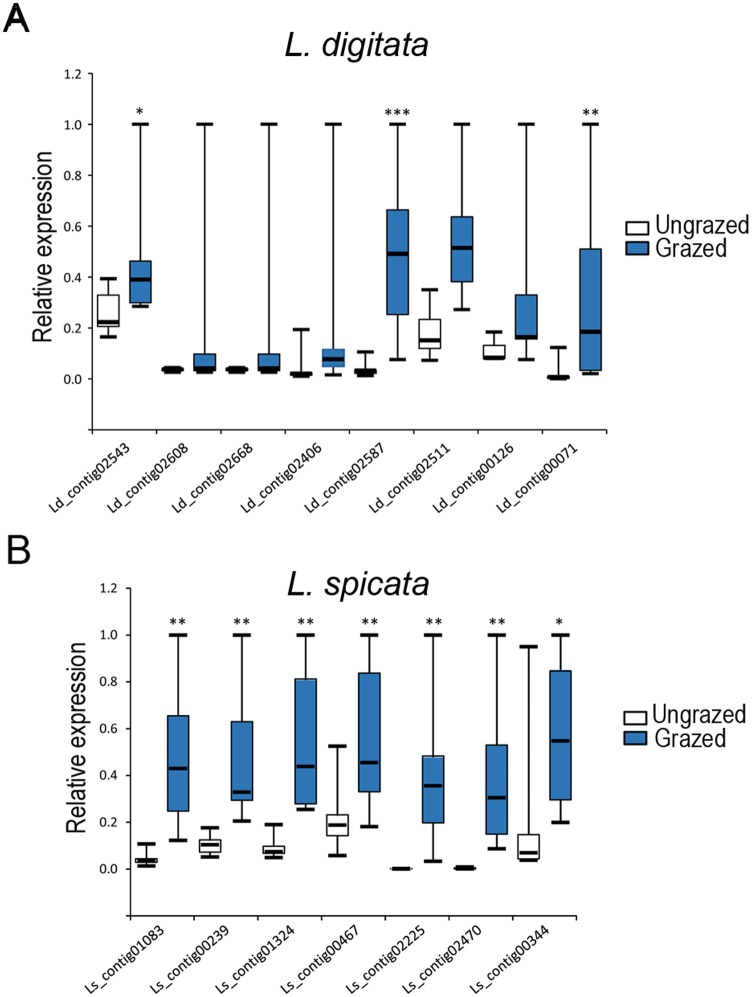
Validation of grazing marker genes in natural kelp populations of *L*. *digitata* and *L*. *spicata*. (**A-B**) Whisker-plot representations of qPCR expression values from candidate grazing marker sequences in ungrazed (white) or grazed (blue) natural populations of *L*. *digitata* and *L*. *spicata* (n = 7–8). Expression values were normalized to a maximum of 1 for each marker. Continous horizontal bars along boxes represent the median values. For each marker, the effect of grazing was tested using U-test (*P<0.05, **P<0.01, ***P<0.001).

We further evaluated whether the identified up-regulated gene sequences could serve as markers of grazing exposure in the field. With this purpose, *L*. *digitata* adult fronds grazed by *Patella pellucida* (S1 Fig) were harvested at the shore of Roscoff—France together with ungrazed individuals as negative controls (n = 7–8). RNA was then extracted from individuals to quantify expression of 8 unigenes (*i*.*e*. Ld_contig02543; Ld_contig02608; Ld_contig02668; Ld_contig02406; Ld_contig02587; Ld_contig02511; Ld_contig00126; Ld_contig00071) that were previously validated in laboratory grazed conditions (Fig 3B). The results showed significant transcript accumulation of only three unigenes, Ld_contig02543, Ld_contig02587 and Ld_contig00071, in grazed adult individuals compared to un-grazed algae collected in the field (Fig 4A).

Similarly, adult fronds of *L*. *spicata* grazed by *S*. *scurra* were harvested at the shore of Las Cruces—Chili (S1 Fig) together with ungrazed individuals as negative controls (n = 7–8). RNA was then extracted from individuals to quantify expression of 7 unigene sequences (Ls_contig01083; Ls_contig00239; Ls_contig01374; Ls_contig00467; Ls_contig02225; Ls_contig02470 and Ls_00344) presenting high confidence up-regulation in laboratory conditions (Fig 3C). All tested unigenes showed significant up-regulation in grazed individuals compared to controls (Fig 4B). Altogether, these results have validated a number of grazing molecular markers in *L*. *digitata* and *L*. *spicata*, demonstrating their strong activation by grazing regardless of developmental stages or laboratory set-up conditions.

## Discussion

Our metabolomic analysis succeeded to distinguish grazed and control metabolomes of *L*. *digitata* and *L*. *spicata* (Fig 1) suggesting these two kelps respond to grazing by their specialist herbivores and regulate their metabolism accordingly. Moreover, metabolite profiling (Fig 2) revealed that grazing induces in *L*. *spicata* early and late responses, involving an early accumulation of aminoacids, followed by the liberation of FFA and the synthesis of prostaglandins.

In *L*. *spicata*, significant accumulations of AAs were detected in grazed individuals during the first six hours of treatment and peaked after 12h. Sulphur containing AAs (SAAs) such as Methionine and its direct precursors Cysteine and Aspartate accumulated rapidly to high levels under herbivore attack. SAAs play key roles in protein synthesis and function, and are also particularly sensitive to oxidation caused by various stresses [19, 24]. Therefore, high accumulation of SAAs could counteract the effect of oxidative stress generated by grazer attack. This hypothesis is supported by previous transcriptome analysis of *L*. *digitata* elicited with oligoguluronates (GGs) [9]. GGs are kelp cell wall digestion products recognized as a microbe-induced molecular pattern (MIMP). *L*. *digitata* associates the presence of GGs with a biotic attack, thereafter generating an oxidative burst cascade resulting in the transcriptional activation of defense responses [6,9]. Results of this analysis identified a Methionine Sulfoxide Reductase (MSR) gene as significantly upregulated. MSRs catalyze the reduction of sulfoxide groups generated in Methionine residues by oxidative stress, and therefore potentially limit loss of protein function due to oxidative damage. The strong accumulation of Glutamate and Proline in grazed *L*. *spicata* likely constitutes additional examples of antioxidant responses. Together with glycine and cysteine, glutamate constitutes a building block of the tripeptide glutathione, a key antioxidant in both biotic and abiotic stress tolerance mechanisms in eukaryotes [25]. Glutathione is used by land plants and brown algae as one of the main antioxidant component in the Ascorbate-Glutathione (ASC-GSH) cycle [26,27]. Beside its function as compatible osmolyte, proline contributes to stabilizing sub-cellular structures (e.g., membranes and proteins), scavenging free radicals thus buffering cellular redox potential under stress conditions in land plants [28]. Moreover, exogenous proline application upregulates the activities of enzymes in the ASC-GSH cycle in plants [29]. It has also been proposed that its accumulation may be part of plant stress signal influencing adaptive responses [28]. Previous studies in *E*. *siliculosus* evidenced a strong accumulation of proline during short-term response to oxidative and osmotic stress [24]. Altogether, the observed increased contents of these AAs are likely to respond to the alteration in the redox cellular status following herbivore attack.

FFA and oxylipins also accumulated in grazed *L*. *spicata*. They followed a delayed dynamics compared to AAs, increasing from 12h to peak at 24h and then decreasing at 48h of grazing. This timing agrees with previous observations of FFA and oxylipin biosynthesis in *L*. *digitata* following abiotic stress conditions [12]. In *L*. *spicata*, grazing induced significant accumulations of both octadecanoid and eicosanoid FFAs together with eicosanoid derived oxylipins. Similar results of FFA and oxylipin accumulations were observed in *L*. *digitata* following bacterial lipopolysaccharide application, which was recognized by the kelp as a Microbe Associated Molecular Patterns (MAMP) [7]. Animals, plants or red algae react to biotic attacks by the activation of signaling reactions which include oxidative bursts and oxylipin formation from either octadecanoid (land plants), eicosanoid (animals) or both (red algae) pathways such as jasmonic acid or prostaglandins [30,31]. In agreement with the functions of FFAs and oxylipins, it was previously shown that arachidonic acid or methyl-jasmonate could trigger an oxidative burst in *L*. *digitata*, which induced resistance against the infection of the endophyte pathogen *Laminariocolax tomentosoides* [15]. In this context, the observed accumulations of FFAs and prostaglandins in *L*. *spicata* in response to grazing likely constitute molecular cues triggering defense reactions against the herbivore *S*. *scurra*. Grazing in *L*. *spicata* induced the synthesis of PGA2, PGB2 and PGJ2 oxylipins that derived from arachidonic acid. In agreement, previous observations in *L*. *digitata* showed that only arachidonic acid but not linoleic acid could trigger defense response against pathogen infection in this kelp [15]. Altogether, these results suggest the importance of eicosanoid-derived metabolites to trigger defense responses during seaweed-herbivores interactions.

Environmental abiotic and biotic factors (such as wounding, herbivore or pathogen attacks) can trigger stress defensive mechanisms in plants through massive reprogramming of gene expression. Our transcriptome analysis, based on the combination of global EST analysis and targeted qPCR validation, attempted to evaluate if a 2-day grazing challenge could induce large gene expression changes in kelps. We detected *in silico* only a limited number of putative differentially expressed unigenes (Fig 3), representing 0.8% of the total identified unigenes. In agreement with the qPCR expression pattern observed for targeted unigenes, these results suggest early transcriptome reprogramming occurring between 6 and 48 hours of grazing pressure. Questions remain if larger differences might occur over longer periods. Similarly, recent transcriptomic analysis of *Fucus vesiculosus* grazed for 3 days by the specialist herbivore, *Littorina obtusata*, revealed only 61 up- and 124 down-regulated genes. A stronger gene regulation was detected between 2 and 3 weeks of grazing (241 and 270 genes differentially expressed at day 15 and 21, respectively) [32]. Moreover, we found very few homologies between our unigene library and known gene functions. This could reflect the low sequence length coverage of the unigene libraries [2], but also partly underlines the paucity of reliable gene annotations for brown algal sequences. Most of the confirmed differentially expressed genes in both kelp species showed a strong peak of induction after 24 and 48 hours of grazing pressure (Fig 3B and 3C), supporting their implication in activation of anti-herbivory adaptive responses. This timing coincides with the observed kinetics of oxylipin synthesis in *L*. *spicata*, suggesting a possible role of oxylipin accumulation for gene upregulation. Nevertheless further experiments are needed to confirm direct activation by FFAs or prostaglandins. Future gain of genomic information will help identifying the functions of these herbivory-responsive genes.

An additional line of evidence supporting the implication of the identified unigenes in grazing response was obtained from our field experiments. This approach was particularly successful in *L*. *spicata*, where the 7 genes validated in laboratory-controlled conditions were significantly upregulated in wound zones created by *S*. *scurra* on fronds collected in the field. The high induction of these genes in recurrently grazed fronds further suggests their association with the setup of persistent responses to herbivory. The observed response was less intense in *L*. *digitata*, with only 3 out of 8 genes showing significant inductions in grazed tissues from natural populations. This discrepancy might point at genes induced only in early developmental stages, as young sporophytes were used for laboratory experiments. It could also indicate genes upregulated very early upon grazing (up to 2 days of grazing) but not later. Indeed the timing of grazing pressure in the field samples was unknown and could therefore exceed 2 days. Recent studies demonstrated the ability of *L*. *digitata* to vehicle systemic signals inducing defense reactions distantly of elicited zones [16]. GG elicitation generated an oxidative burst signal that was transmitted systemically through the kelp tissue, activating defense genes in a process that likely involves oxylipins signaling. Moreover, authors observed reduced grazing activity of *P*. *pellucida* distantly from elicited areas. These results are reminiscent of recent findings in land plants showing propagation of jasmonate signaling triggering defense distantly of wounded areas [33]. In line with these results it would be interesting to assess if the identified unigenes can be activated by oxylipins signaling pathways distantly of grazed areas.

## Conclusion

Using laboratory-controlled bioassays we have shown that grazing by specialist herbivores induces both significant modification of the metabolome and specific gene regulation in the two kelp species *L*. *digitata* and *L*. *spicata*. In addition, metabolite profiling shows that *L*. *spicata* reacts to herbivore attack by a rapid response following the first hours of challenge consisting in the accumulation of AAs likely constituting an antioxidant response. This early response is followed by a secondary reaction consisting in the synthesis of FFAs and oxylipins, which may act as signaling cues to trigger latter immune responses against herbivores. Finally, our results determined a set of genes related to grazing pressure in field populations of marine kelps. These genes could be used as markers to study the functional dynamics of seaweed-herbivore interactions in natural populations or as selection markers *i*.*e*. in the context of kelp breeding programs. Recent developments in next generation sequencing technologies open exciting perspective of this work. Genome Wide Association Studies of grazed kelp populations might provide more precise traits of evolution related to herbivore defense in these organisms.

## Supporting information

S1 Fig Representative pictures of kelps with their respective specialist herbivores. (A) *Laminaria digitata* and *Patella pellucida* Linnaeus. (B) *Lessonia spicata* and *Scurria scurra*, on Algal tissues showing typical grazing damages are presented. Photos by LC, FT, CF, SF and CL.
(TIF)

S1 Table Primers used in the qPCR analysis study. (XLSX)

S2 Table Detailed list of identified ions by LC/MS analysis and their relative abundance from *Laminaria digitata* samples, under control and grazed conditions, collected after 6, 12, 24 and 48h (n = 4, except for Grazed-48h n = 2). Peak assignation, ion identification and relative abundance calculation were processed by XCMS analysis. Each ion is identified by a unique ID (MxTy) indicating its mass (Mx, mz) and retention time (Ty, min).
(XLSX)

S3 Table Detailed list of identified ions by LC/MS analysis and their relative abundance from *Lessonia spicata* samples, under control and grazed conditions, collected after 6, 12, 24 and 48h (n = 3, except for Control-48h n = 2). Peak assignation, ion identification and relative abundance calculation were processed by XCMS analysis. Each ion is identified by a unique ID (MxTy) indicating its mass (Mx, mz) and retention time (Ty, min).
(XLSX)

S4 Table Statistical analysis comparing ion relative abundances of Grazed vs. Control *Laminaria digitata* samples, collected after 6, 12, 24 and 48h (n = 4, except for Grazed-48h n = 2). Peak assignation, ion identification and relative abundance calculation were processed by XCMS analysis. Each ion is identified by a unique ID (MxTy) indicating its mass (Mx, mz) and retention time (Ty, min). Ion ID indicated in bold shows T-test and FDR tests with *P*<0.01.
(XLSX)

S5 Table Statistical analysis comparing ion relative abundances of Grazed vs. Control *Lessonia spicata* samples, collected after 6, 12, 24 and 48h (n = 3, except for Control-48h n = 2). Peak assignation, ion identification and relative abundance calculation were processed by XCMS analysis. Each ion is identified by a unique ID (MxTy) indicating its mass (Mx, mz) and retention time (Ty, min). Ion ID indicated in bold shows T-test and FDR tests with *P*<0.01.
(XLSX)

S6 Table Fold-Change and statistical analysis comparing amino acid, free fatty acids and oxylipin relative abundances of Grazed vs. Control *Lessonia spicata* samples, collected after 6, 12, 24 and 48h. For each metabolite and sampling time, fold-change was calculated by dividing the average of ion relative abundance in Grazed samples by that from Control samples (n = 3, except for Control-48h n = 2).
(XLSX)

S7 Table List of 122 putative differentially expressed unigenes under grazing pressure in *Laminaria digitata*, identified by 454 sequencing and EST analysis. (XLSX)

S8 Table List of 132 putative differentially expressed unigenes under grazing pressure in *Lessonia spicata*, identified by 454 sequencing and EST analysis. (XLSX)

S9 Table Fold-Change and statistical analysis comparing qPCR transcript levels of selected unigenes between grazed and control individuals of *L*. *digitata*. (XLSX)

S10 Table Fold-Change and statistical analysis comparing qPCR transcript levels of selected unigenes between grazed and control individuals of *L*. *spicata*. (XLSX)
