# Supplementary figures and images for: Herbivore-induced chemical and molecular responses of the kelps Laminaria digitata and Lessonia spicata
Source: PLoS One. 2017 Mar 2;12(3):e0173315. doi: 10.1371/journal.pone.0173315 (PMC5333891; doi:10.1371/journal.pone.0173315)

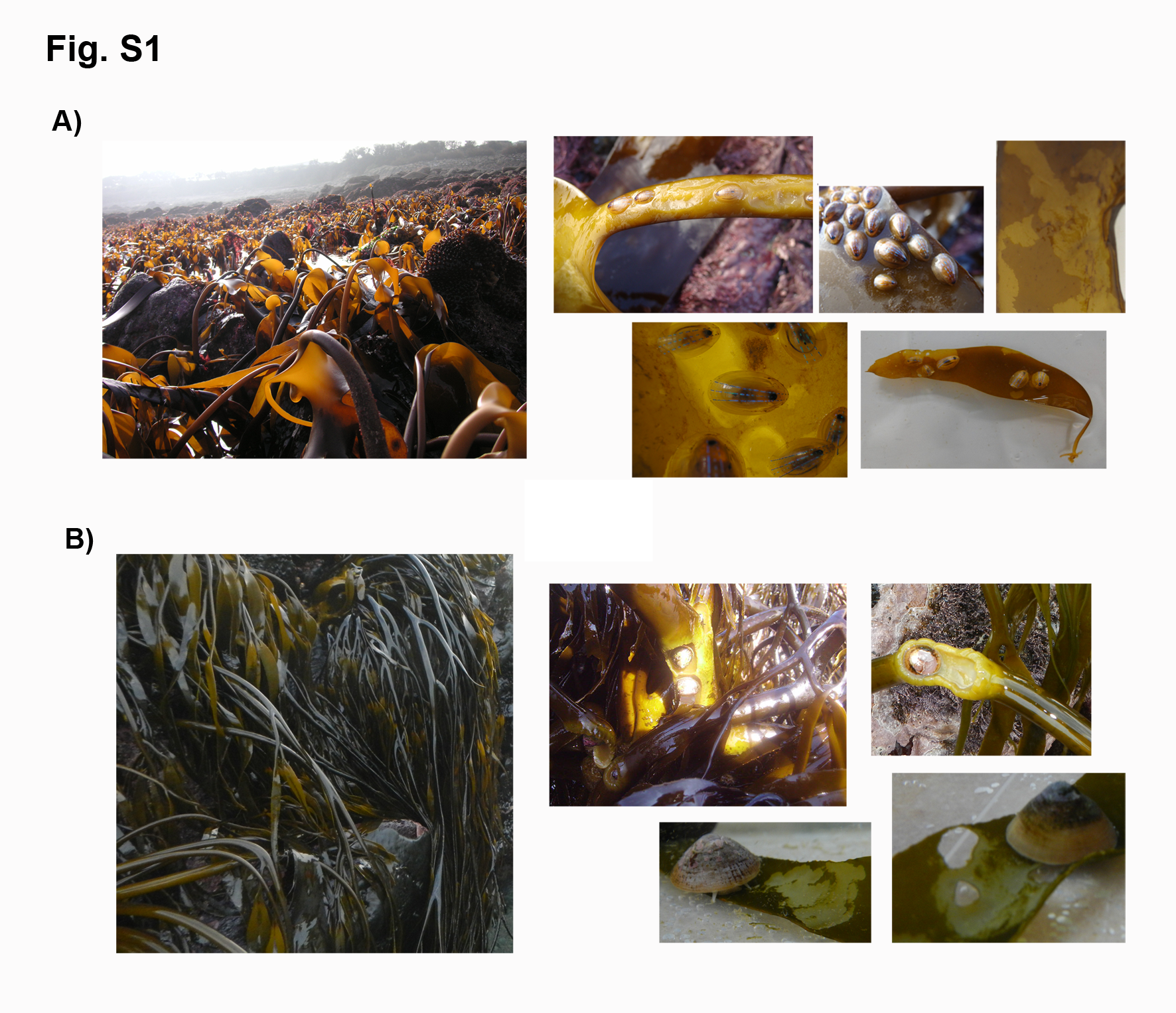

Supplement: S1 Fig — (A) Laminaria digitata and Patella pellucida Linnaeus. (B) Lessonia spicata and Scurria scurra, on Algal tissues showing typical grazing damages are presented. Photos by LC, FT, CF, SF and CL. (TIF) [file pone.0173315.s001.tif]
